# Chimpanzees’ understanding of social leverage

**DOI:** 10.1371/journal.pone.0207868

**Published:** 2018-12-12

**Authors:** Alejandro Sánchez-Amaro, Shona Duguid, Josep Call, Michael Tomasello

**Affiliations:** 1 Department of Cognitive Science, University of California San Diego, San Diego, United States of America; 2 Department of Developmental and Comparative Psychology, Max Planck Institute for Evolutionary Anthropology, Leipzig, Germany; 3 Warwick Business School, University of Warwick, Warwick, United Kingdom; 4 School of Psychology and Neuroscience, University of St. Andrews, St. Andrews, United Kingdom; 5 Department of Psychology and Neuroscience, Duke University, Durham, United States of America; Rice University, UNITED STATES

## Abstract

Social primates can influence others through the control of resources. For instance, dominant male chimpanzees might allow subordinates access to mate with females in exchange for social support. However, little is known about how chimpanzees strategically use a position of leverage to maximize their own benefits. We address this question by presenting dyads of captive chimpanzee (*N* = 6) with a task resulting in an unequal reward distribution. To gain the higher reward each individual should wait for their partner to act. In addition, one participant had leverage: access to an alternative secure reward. By varying the presence and value of the leverage we tested whether individuals used it strategically (e.g. by waiting longer for partners to act when they had leverage in the form of alternatives). Additionally, non-social controls served to show if chimpanzees understood the social dilemma. We measured the likelihood to choose the leverage and their latencies to act. The final decision made by the chimpanzees did not differ as a function of condition (test versus non-social control) or the value of the leverage, but they did wait longer to act when the leverage was smaller—particularly in test (versus non-social control) trials suggesting that they understood the conflict of interest involved. The chimpanzees thus recognized the existence of social leverage, but did not use it strategically to maximize their rewards.

## Introduction

In bargaining with others, a major source of power or leverage for the individual is access to alternatives [1, 2]. Access to alternatives means that the individual does not need the partner as much as the partner needs him and this creates an asymmetry of power independent of any pre-existing dominance relations. In market relationships, for instance, a buyer can persuade a vendor to lower prices if the buyer can acquire the same product at a lower price on the next stall. However, the opposite can occur when the vendor is the only one that can provide the good. In this case the vendor can use this opportunity as bargaining leverage to raise prices.

To understand such examples, researchers have developed controlled experiments to assess how human adults might take advantage of leverage in social interactions. In particular, using game theory models [3, 4], researchers have recreated plausible social dilemmas to investigate whether human adults use leverage strategically to maximize incomes in bargaining situations. The introduction of leverage—for instance, in the form of alternative options for one of the interacting partners—automatically creates an asymmetry between participants.

Experiments have explored how the introduction of leverage affects individual decisions during bargaining interactions [5]. For instance, Cooper and colleagues [6, 7] investigated whether outside options for one player in a Battle of the Sexes game (BOS) would help players to coordinate. In a BOS game each player has two options: Option A which is preferred for player A (e.g. watch a Sci-Fi movie) and option B which is preferred for player B (e.g. watch a Western movie). Importantly, players must choose the same option (either both A or both B). Otherwise they both lose (e.g. none of them wants to go to the movies alone). Thus, while both players prefer the other to choose their preferred option, it is still beneficial to coordinate on the same option. In the study by Cooper and colleagues Player A had the opportunity to play the BOS game (to either get 600 or 200 tickets to win a lottery, depending on their choice and their partner’s choice) or choose an alternative option that gave both players A and B an intermediate reward (300 tickets). Experimenters found that only 20% of those in the player A position chose the alternative option. Instead, a majority played the BOS and chose the option that would maximize their own rewards (600 tickets for them and 200 tickets for player B). At the same time, those in the position of player B (those without the alternative option) used forward induction to predict the decisions of player A. This resulted in players coordinating on the most favorable outcome for Player A in about 90% of decisions. In other words, player A used their leverage strategically while players B understood this position of leverage and adapted their decisions to obtain a share of the rewards. Similar effects of leverage have been observed in other common games such as the Ultimatum Game [8–10].

These types of asymmetric relationships with positions of leverage are also found among social non-human primates [11, 12]. For example, among group-living primates, different external commodities such as social support or the knowledge of an expert may be in more demand than others, such as grooming. According to biological markets theory, human and non-human primates exchange these commodities in accordance with the laws of supply and demand [13–15]. If so, individuals in possession of the most valuable commodities are in a position of leverage over others. Both field observations [11] and experimentally controlled studies [16] provide examples showing how individuals might benefit from leverage through the use of certain commodities or resources. In a recent study, Fruteau and colleagues [16] trained a middle rank female vervet monkey to open a box that delivered food to the whole group. Over time, this female received increased amounts of grooming from other group members, perhaps in exchange of the food that she indirectly provided them. When a second female was also trained to open the box, the first female lost part of her leverage and, in turn, received less grooming.

Among chimpanzees, we also find examples in which certain individuals might benefit from being in a position of leverage. Duffy and colleagues [12] recently found that dominant males granted subordinate males the opportunity to mate with females in exchange of social support. However, from these examples it is difficult to know whether individuals intentionally use their leverage to obtain the most from their social interactions. In other words, it remains unknown whether non-human primates such as chimpanzees understand the role of leverage as a way to maximize their benefits.

To address these questions, we investigated the strategies chimpanzee dyads would use in a social dilemma task where one of the participants (henceforth the subject) was in a position of leverage over the other (henceforth the partner). Based on a previous study investigating the strategies chimpanzees and five-year old children use to solve a conflict of interest [17], we presented chimpanzees with a task (in the form of a rotating tray) that always resulted in an unequal reward distribution. In this scenario, the best strategy for each chimpanzee was to wait for the other to pull a rope connected to one end of the tray. This provided the individual that pulled with one reward and the individual that waited with three rewards. In total, apes had a maximum of 20 seconds to retrieve the rewards. In addition, both participants were also tested alone (non-social control trials) to assess whether they understood that the conflict of interest was only present in the social interaction.

We added leverage to this task by providing an alternative option for subjects only. The alternative option consisted of one of three possible food rewards on the subjects’ alternative platform:

1) *No alternative* condition: There was no food baited on the subjects’ alternative platform. In this condition, the game was symmetrical. Thus, we expected no differences between subjects and partners: both individuals should wait for each other to pull from the rotating tray during test trials (when both individuals are present). During non-social trials subjects and partners should pull from the rotating tray without waiting.
2) *Leverage* condition: The subject’s alternative platform contained two banana slices. In this condition, subjects had potential leverage over partners. Subjects had the option to obtain two rewards baited on their alternative platform rather than pulling (providing one reward for themselves and three for the partner). If subjects exerted leverage to obtain the maximum reward, we expected them to wait for their partners to pull from the rotating tray before making the decision between sliding the door open for the rotating tray or alternative platform during test trials. During non-social control trials we expected subjects to maximize their rewards by accessing the two banana slices in the alternative platform.
3) S*ecure alternative* condition: The subject’s alternative platform contained four banana slices. In this condition four is more than can be obtained from the rotating tray thus, we expected subjects to access the alternative platform regardless of their partner’s decision or whether it was a test or non-social control trial.

## Material and methods

### Subjects

We tested 6 captive chimpanzees (1 female; M_age_ = 14.6 years) housed at the Wolfgang Koehler Primate Research Center in Leipzig zoo (Table A in S1 File for more information about the apes that participated in the study). During the first phase of the study, the chimpanzees made up 3 unique pairs. In the second phase pairs were reshuffled to create 3 new pairs.

Pairings were pseudo-randomly assigned based on their degree of social tolerance and the limited number of individuals we could test (N = 6). The only female, Taï, was paired with a subadult male and with a mid-ranking male to minimize the effect that dominance might have.

### Ethics statement

The study was approved by an internal ethics committee at the Max Planck Institute for Evolutionary Anthropology. The study complies with the Weatherfall report “The use of non-human primates in research”. The study also complies with the ‘EAZA Minimum Standards for the Accommodation and Care of Animals in Zoos and Aquaria’, the ‘WAZA Ethical Guidelines for the Conduct of Research on Animals by Zoos and Aquariums’ and the ASAB/ABS ‘Guidelines for the Treatment of Animals in Behavioural Research and Teaching’. IAUCUC approval was not necessary to conduct this research. Chimpanzees were housed in large semi-natural indoor and outdoor enclosures and the research was conducted in their sleeping rooms. Apes had regular feeding schedules, daily enrichments and water ad libitum. Apes were never food- or water-deprived and could voluntarily participate in the test by entering their sleeping rooms. During the test sessions chimpanzees had access to water *ad libitum*.

### Materials

**Fig 1:**
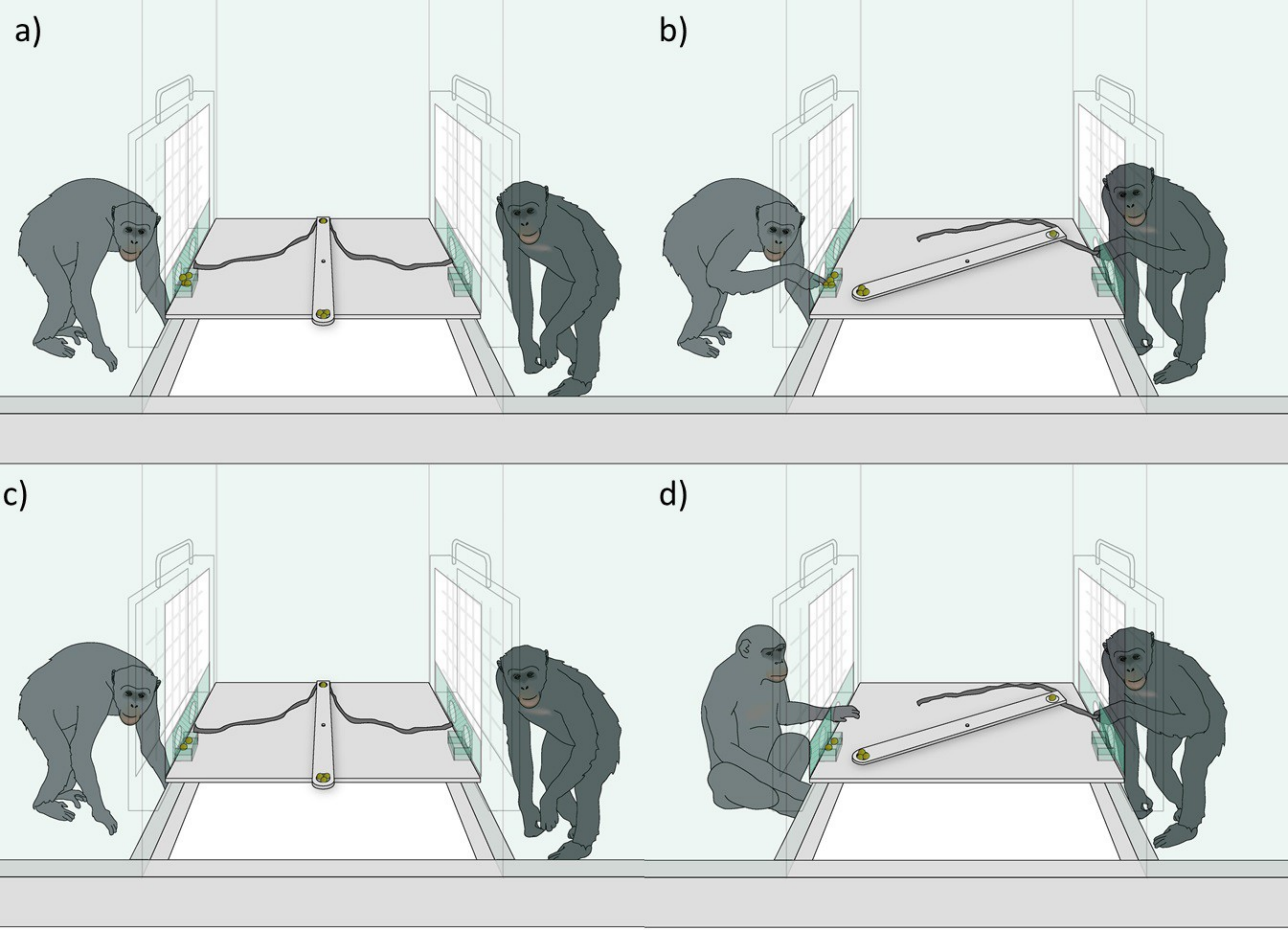
Study apparatus. Figures a) and b) depict the subject (chimpanzee on the left) with four rewards on the alternative platform, with the subject accessing the alternative platform in Figure b). Figures c) and d) depict the subject with two rewards on the alternative platform, with the subject accessing the rotating tray once the partner has started to pull in Figure d).

We tested pairs of chimpanzees with an apparatus placed between two adjacent test rooms. The apparatus consisted of a narrow rotating tray (10x91 cm) attached to the center of a larger platform (88.5x96.5 cm). The tray could rotate 360° on the horizontal plane. In addition, two alternative platforms (10X10 cm) were located on the exterior corners of the main platform (Fig 1), one accessible from each room.

Chimpanzees faced each other across the apparatus from separate rooms. Each individual could access either the rotating tray or their alternative platforms by sliding a door to the left or to the right. If they slid the door to the right they could pull from Velcro ropes (76 cm) that were attached to the interior end of the rotating tray. When a subject pulled its rope, the interior end of the tray rotated towards her while the free end rotated towards the partner. If they slid the door to the left they could directly access their alternative platform. When a subject slid the door to either side, a locking mechanism prevented the door from returning to its original position. In other words, subjects could only make one choice per trial.

### Design and procedure

Prior to test sessions all subjects completed three individual training phases.

#### Training phase 1

This first training phase served to show chimpanzees that they could access the rewards either from the rotating tray or from the alternative platform, and that they would experience 10 seconds delay between experimenters providing access to each sliding door. By letting chimpanzees access the rewards from both sides of the apparatus we expected them to infer that other individuals could potentially obtain those rewards in the future (i.e. in test trials when partners are located on the other side of the apparatus).

In two of the four sessions that made up this training phase, individuals experienced two trials with the interior end of the rotating tray baited with food and two trials with the alternative platform on their side baited. To succeed, chimpanzees had to access the baited food location.

In the remaining two sessions, chimpanzees experienced four trials with food baited on both alternative platforms. The door connecting the two test rooms remained open to allow individuals to retrieve the rewards from both alternative platforms. In two trials an experimenter allowed chimpanzees to access both baited platforms at the same time (simultaneous trials). In the other two trials, chimpanzees accessed the rewards sequentially—with a ten-second delay between the openings of both sliding doors (sequential trials). During simultaneous trials, chimpanzees could retrieve the food from the closest alternative platform via the sliding door and then immediately move to the adjacent room to retrieve the rewards from the second platform. Sequential trials were the same as simultaneous trials except that, once chimpanzees were in front of the second door, they had to wait ten seconds until the experimenter allowed them access to the second platform. In this way, individuals experienced the delay between the removals of the two blocking pegs as would be the case for subjects and partners during test trials. All the baited rewards consisted of a single banana slice.

We alternated the presentation of the two types of session; individuals changed their starting location (either from the left or the right test room) between sessions. All individuals, except two, performed four training sessions; the remaining two needed a fifth session to reliably access the food.

#### Training phase 2

In the second training phase both the rotating tray and one alternative platform were baited. This training phase was designed to provide chimpanzees the opportunity to learn how to access the highest number of rewards.

On half of the trials the rotating tray was baited with two banana slices on each end—for a total of four—while the alternative platform contained only two banana slices. In these trials, apes should access the rotating tray. On the other half of the trials there was only one banana slice on each end of the rotating tray and four banana slices on the alternative platform. In these trials, apes should access the alternative platform. The door connecting the two test rooms remained open to allow individuals to retrieve the rewards from both ends of the rotating tray.

To begin the trial, an experimenter removed the peg blocking the first sliding door. The experimenter removed the peg blocking the second sliding door ten seconds after chimpanzees accessed their alternative platform or, when individuals accessed the rotating tray, the experimenter removed the peg blocking the adjacent door ten seconds after chimpanzees positioned themselves in front of it.

Chimpanzees received sessions consisting of four trials of each type in a randomized order. On each session, chimpanzees started the trials from one side of the apparatus and changed sides between sessions. To succeed in this training phase and proceed with the last training phase, individuals had to choose the option that maximized their rewards in at least 80% of trials in two consecutive sessions. Chimpanzees required between 2 and 16 sessions to reach the criteria (Med = 7).

#### Training phase 3

In the two previous training phases individuals could always obtain all the rewards baited on both sides of the rotating tray—something that was not possible during test trials. Thus, the third training phase served to highlight that chimpanzees could not always obtain all the rewards baited on the rotating tray.

For this purpose, we presented individuals with the same two types of trials as in the previous training phase (rotating tray and alternative trials) while we manipulated whether the door connecting the two test rooms remained open or closed. This resulted in four different trial combinations (highest value reward on the rotating tray or the alternative platform and the connecting door open or closed). The correct response then depended not only on the reward distribution but also on which sliding door could be accessed: when there were two food items on either end of the rotating tray and two on the alternative platform, the rotating tray should only be preferred when the door connecting both test rooms was open. With the door closed both options were the same (they chose the platform in 55% of trials). When there were four items on the alternative platform, this was always the correct choice. During trials with the connecting door closed, the experimenter removed the peg blocking the second sliding door ten seconds after individuals acted. During trials with the connecting door open, the experimenter removed the peg blocking the second sliding door ten seconds after chimpanzees positioned themselves in front of it.

Each individual received a minimum of four training sessions. Each session was composed of two trials per condition for a total of eight trials. Chimpanzees changed their starting location between sessions. As in previous training phases, individuals succeeded if they maximized their rewards. All chimpanzees except one performed the four training sessions without errors before they proceeded with the test sessions. The remaining chimpanzee performed a fifth session before the test sessions.

#### Test sessions

Within a given pair, individuals were randomly assigned the role of subject or partner. Only subjects could access food rewards baited on their alternative platforms. There were three conditions depending on the amount of food baited on the alternative platforms: *no alternative*, *leverage* and *secure alternative* condition. Partners could also access their alternative platforms despite being empty. Both individuals could choose to access the rotating tray. This was always baited with one banana slice on the interior end (where the rope was attached) and with three slices on the exterior end.

The subject and partner did not access the apparatus at the same time; one individual (either the partner or the subject) always started the trial ten seconds before the other. The delay between individuals’ access to the apparatus gave us the possibility to measure whether chimpanzees made decisions in anticipation of partners’ behaviour. The trial was initiated by the removal of a central peg in the sliding door by the experimenter, allowing the subject or the partner to open its sliding door. That chimpanzee had ten seconds to decide whether to slide the door—to either access its rope or its alternative platform—or to wait. At which point, a second experimenter removed the central peg blocking the sliding door of the second participant. After a further ten seconds later, one of the experimenters removed all the remaining rewards and the trial ended.

Each pair was tested together for a total of 16 test sessions. After eight sessions, pairs exchanged subject-partner roles. Each individual was the first to access the apparatus in half of the sessions (four sessions as subject and four as partner), the pairs alternated the starting order between sessions. We changed roles after eight sessions instead of between sessions to facilitate the learning and understanding of each individuals’ role. In our statistical analysis we have controlled for this.

Each test session was composed of three test and three non-social control trials. During test trials both the subject and the partner were present. During non-social control trials only the individual acting first on that session could access the apparatus while the second individual waited one room away from the apparatus (though still visible to the individual with access to the apparatus). The two sets of trials were presented in blocks within a session (i.e. the three test trials followed by the three non-social control trials or vice versa). Within a block there was one trial of each leverage condition. The presentation order of the blocks varied between sessions and the trial order within blocks was randomized. Between sessions individuals changed the side from which they accessed the apparatus (from either the left or the right test room).

#### Coding

Our overall measure of success was the percentage of trials in which at least a member of the pair obtained a reward. To study chimpanzees’ behaviour we focused on their choices and latencies to act. Chimpanzees had three different choices: a) access the rotating tray b) access the alternative platform and c) no action.

We measured the latency to open the sliding door (i.e. latency to make a choice) and the latency to pull the rope. The latency to open the sliding door was the time elapsed from the experimenter removing the peg until the subject slid the door to one side. In other words, until the moment they could no longer change their decision. The latency to pull was the time from the subject sliding the door to access the rotating tray until the subject pulled the rope. From the partner’s perspective, we measured the total time from removing the peg until the partner pulled the rope. Partners almost never accessed the alternative platform (< 1% of trials) which was always empty.

### Statistical analysis

All the analyses were run using R statistics (version 3.1.1). We used a generalized linear mixed model (Model 1) to investigate subjects’ binary choices—to either access the alternative platform or the rotating tray. We used mixed-effects Cox proportional hazards models (Models 2 to 4) to analyze subjects and partners latencies to act. The results of Models 2 to 4 are reported as hazard ratios (HR). An HR greater than one indicates an increased likelihood of acting (either opening the door in model 2, or pulling in models 3 and 4), and an HR smaller than 1 indicated a decreased hazard of acting. In addition, to obtain the p-values for the individual fixed effects we conducted likelihood-ratio tests. See S1 File for details on the model analysis.

## Results

Overall, dyads obtained rewards in 78% of trials. Subject chimpanzees accessing the apparatus first (ten seconds before their partner), did not strategically use their leverage to maximize their rewards (i.e. when the leverage consisted of two rewards they did not access the rotating tray after the partner had pulled). Our analysis of the factors influencing their choices found a main effect of leverage (GLMM: *χ*^2^ = 14.24, df = 1, p<0.001, N = 288); reflecting the significant tendency to choose the food baited on the alternative platform for themselves whenever it was more than was directly available on the rotating tray. In other words, they chose the alternative platform in over 92% of trials when it contained two or four rewards (see Table 1). The likelihood to choose the alternative platform did not vary between test and non-social control trials (GLMM: *χ*^2^ = 1.14, df = 1, p = 0.28, N = 288).

**Table 1:** Percentage of trials that subjects and partners access the rotatory tray across each combination of the study conditions.

	Subjects		Partners	
	Test trials (%)	Non-social controls (%)	Test trials (%)	Non-social controls (%)
no alternative	99	94	96	92
leverage	26	8	83	90
secure alternative	10	0	85	92

**Fig 2:**
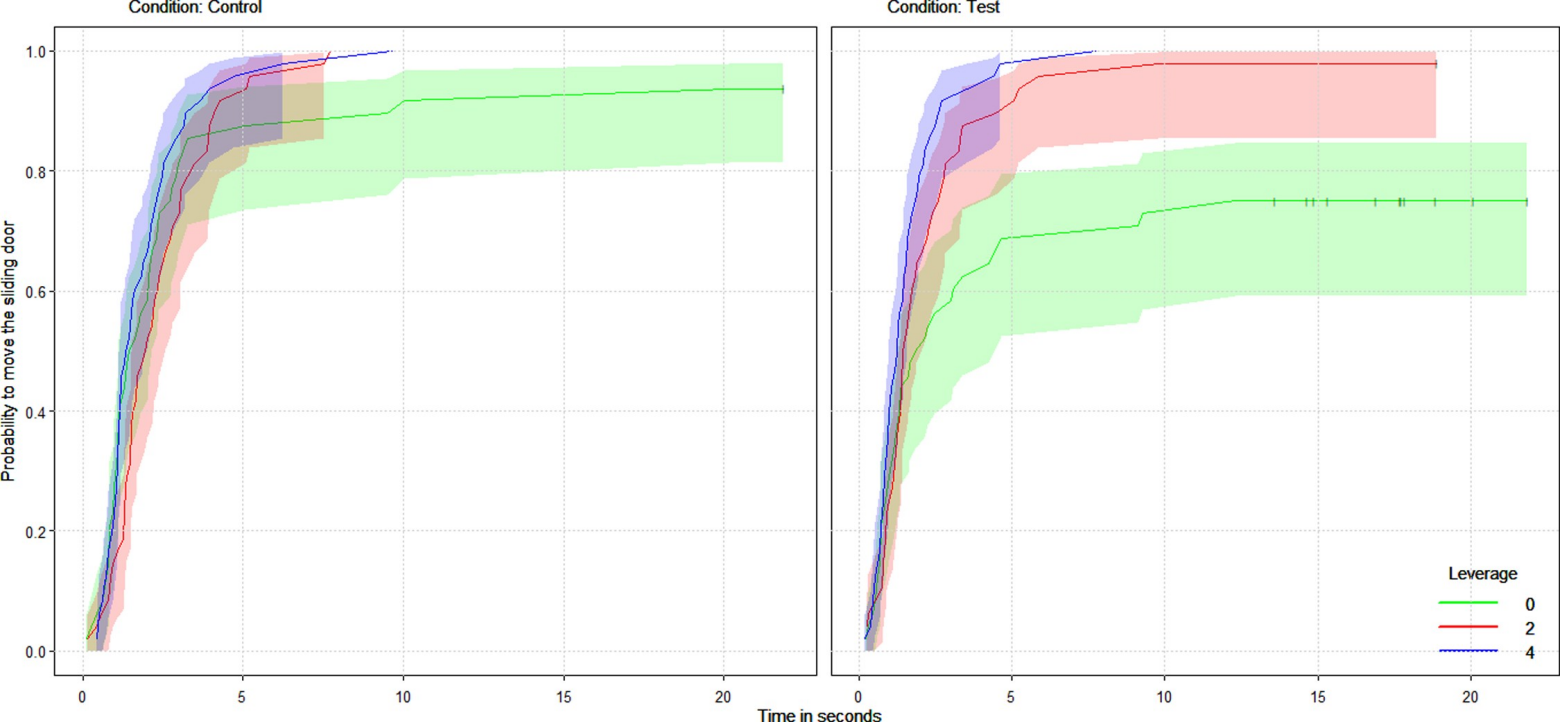
Survival curve of the probability of chimpanzee subjects to move their sliding door across time as a function of the condition (non-social control trials on the left side and test trials on the right side) and the food presented on the alternative platform. A lower probability of acting (e.g. in test trials with leverage level zero) means that chimpanzees waited longer to open their sliding door. The horizontal whiskers represent the censored-data (the events that were terminated either by the partner acting before them or by the experimenter). Chimpanzees waited longer when there was a partner and no food they could access without the partner.

In contrast to subjects’ choices, when we consider the latencies to open the sliding door (either to the rotating tray or the alternative platform), we found that both the condition (test versus non-social control trials) and the amount of food baited on the alternative platform had a significant effect on subjects’ latencies to open the sliding door. In particular, we found that the they acted more slowly the smaller the amount of food on the alternative platform during test trials but in non-social control trials the food baited on the alternative platform did not seem to affect subjects’ latencies to move the sliding door (two-way interaction between condition and leverage: coxme, HR = 1.45, p = 0.004, N = 288, CI = 1.12, 1.87, see Fig 2).

**Fig 3:**
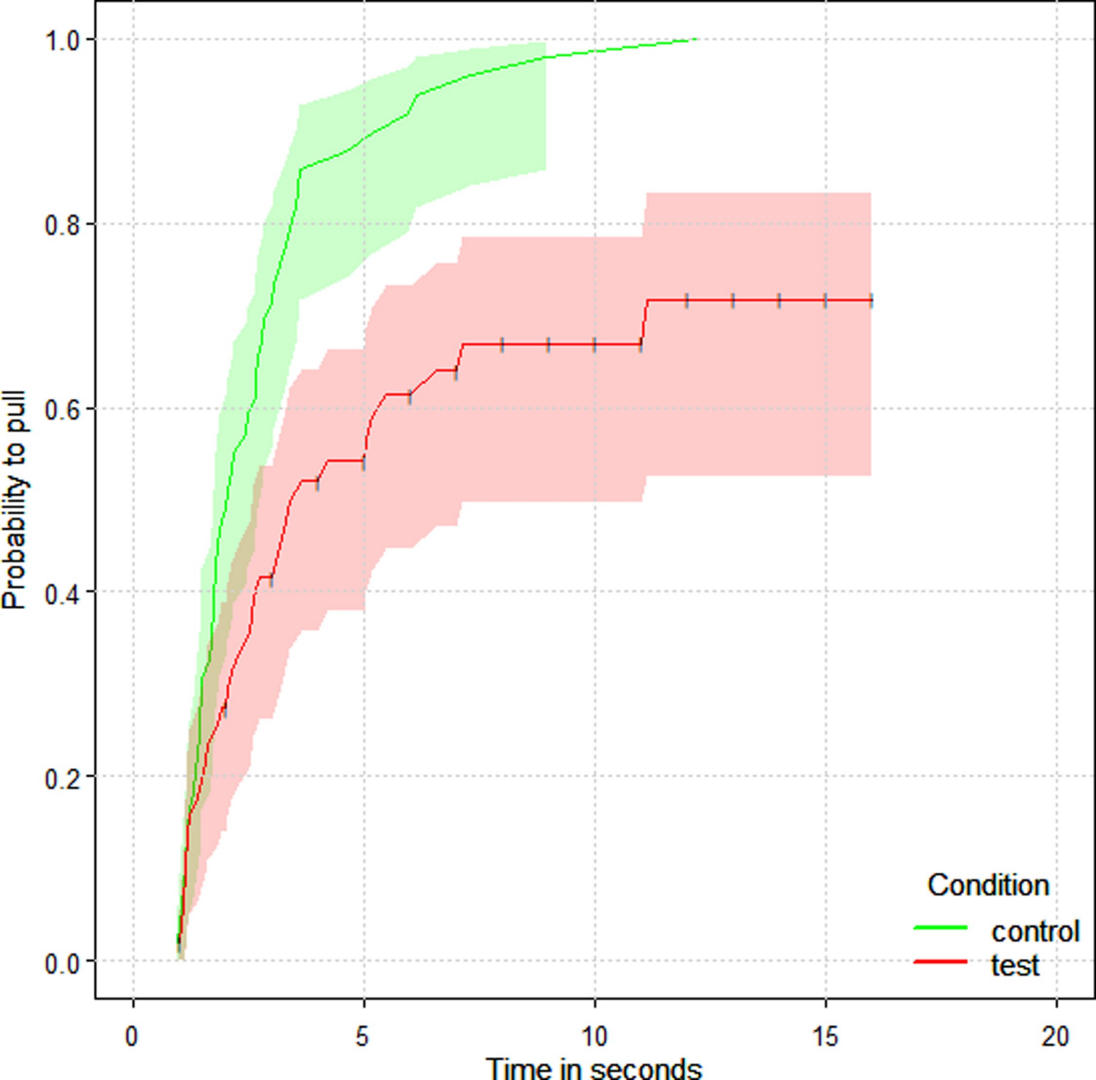
Survival curve of the probability of chimpanzee subjects to pull from their rope across time as a function of the condition presented (non-social control trials in green and test trials in red). A lower probability of acting means that chimpanzees waited longer to pull from their rope. The horizontal whiskers represent the censored-data (the events that were terminated either by the partner acting before them or by the experimenter). Chimpanzees waited longer to pull in the presence of a partner.

We further investigated understanding of the conflict of interest and the potential actions of the subject by looking at the latency to pull the rope after accessing the rotating tray via the sliding door. We found that subjects waited significantly longer during test compared to non-social control trials (coxme, HR = 0.36, p < 0.001, N = 101, CI = 0.22, 0.58). In other words, when the partner was present and could potentially pull the rope, the subject waited longer. This suggests that when chimpanzees were in the subject role they understood the conflict of interest: by waiting longer to pull when a partner was present, subjects increased their chances to get the large food rewards baited on the exterior end of the rotating tray (Fig 3).

**Fig 4:**
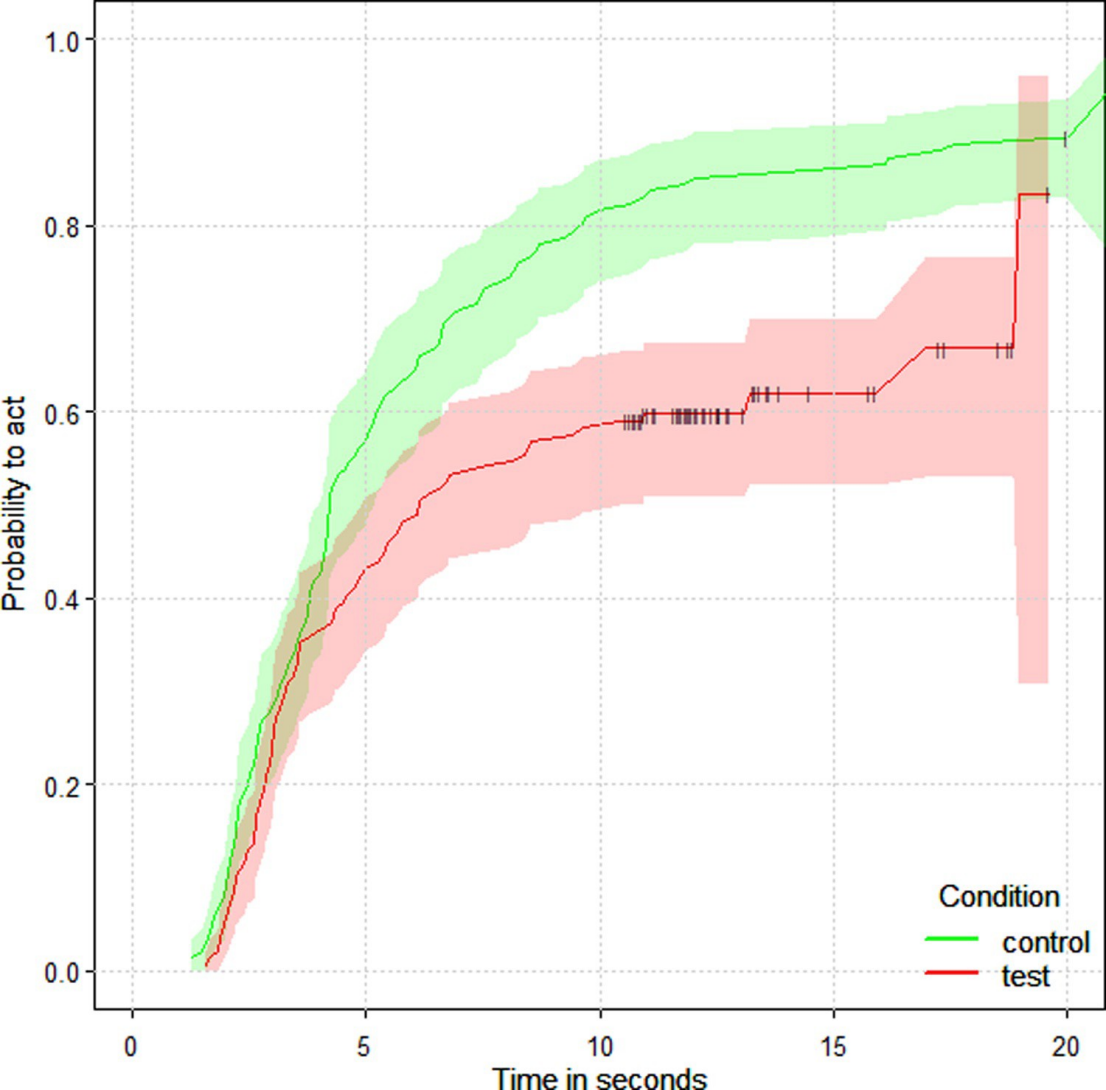
Survival curve of the probability of partners to act (i.e. move the sliding door and pull from their rope) across time as a function of the condition (non-social control trials in green and test trials in red). A lower probability of acting means that chimpanzees waited longer to open their sliding door and pull. The horizontal whiskers represent the censored-data (the events that were terminated either by the subject acting before them or by the experimenter after 20 seconds). Partners waited longer to pull in the presence of a subject.

Finally, we also investigated whether chimpanzees in the role of partners changed their latency to act between test and non-social control trials and the amount of food baited on the subjects’ alternative platform. We analyzed the latency between having access to the apparatus (when the pin was removed) and the moment they pulled the rope (partners chose to access the alternative platform in only 0.9% of all trials). As with the subjects, we found that when chimpanzees were in the partner role they waited significantly longer to pull in test compared to non-social control trials (coxme, HR = 0.47, p = 0.002, N = 280, CI = 0.29, 0.76; Fig 4). Interestingly, the latencies did not depend on food baited on the subjects’ alternative platform. Together, these results support those from the subject role that the chimpanzees understood the conflict of interest presented by the rotating tray, but there was no indication that partners considered subjects’ alternatives. See S1 File for more model details.

## Discussion

By creating an asymmetrical conflict of interest between two chimpanzees we could investigate how chimpanzees resolved this conflict of interest and whether they would use a position of leverage to increase their own gains. We found that the chimpanzees failed to use the leverage as a means to maximize their rewards but their waiting times did differ according to the value of their leverage.

If the chimpanzees were using the leverage (in the form of an alternative option) to maximize their rewards we would have expected them to keep both options open by refraining from choice until the partner conceded to pull for the one reward and provide the subject with three rewards. Instead, the final decisions of chimpanzees in the subject role was to choose the alternative platform baited with two rewards on a large majority of trials (92%), irrespective of the partner’s presence, rather than use it effectively as leverage. Consequently, chimpanzees in the role of subjects never pulled from the rope before their partners either. These results are in line with results on other primate social dilemmas. For instance, in the Ultimatum Game chimpanzees tend to maximize their rewards whenever possible [18] (but see [19]). In this game a donor has the opportunity to give a receiver any fraction of a total sum of rewards as an offer. If the receiver accepts the offer, the amount is divided accordingly. If the receiver rejects the offer, both the receiver and the donor lose all rewards. As in our study, partners accept the less preferred reward if that is the only option they can get. Similar results in coordination games such as the Assurance and the Snowdrift game suggest that some individuals seem to be satisfied with any share of the rewards [17, 20] regardless of what others obtain.

However, a closer look at the behavior found that the latencies to make the decisions showed that subjects (accessing the apparatus first) did distinguish between different amounts of food baited on their alternative platform. Importantly, this was only the case in test trials and not during non-social control trials. Therefore, by waiting differently depending on their amount of food baited on their alternative platform and the partner’s presence, chimpanzee subjects clearly acted in ways that increased their likelihood to maximize rewards (see [17, 21, 22] for similar results in other social dilemmas). In addition, once the subject or partner had opened the sliding door to access the rotating tray, they also waited longer to pull during test trials in comparison to non-social control trials. The addition of these non-social control trials in the test advances our understanding of chimpanzees’ decision-making strategies to resolve conflicts of interest. Previous studies found that chimpanzees waited strategically for others to act when individuals’ preferences were not aligned [17, 21]. In the current study, the contrast between non-social controls and test trials provides further evidence that chimpanzees understood the social nature of the conflict by waiting longer to act when their partners were present. These findings (i.e. that chimpanzees may consider others’ decisions before acting) support recent observations suggesting that chimpanzees might wait for specific individuals to start chases during cooperative hunts [23, 24].

A full understanding of the leverage utility would require chimpanzees to make inferences about others mental states (i.e. subjects and partners had to infer about the decision-making of the other with regard to each others’ options). Recent evidence of chimpanzees’ perspective-taking abilities in other social scenarios [25, 26] gives support to this possibility. However, in the current study, strategic decision-making (i.e. decisions aim to maximize their own gains while considering the potential decisions of others) was only reflected in the latencies to act, but not in the final decisions. Chimpanzees in the subject role hardly ever waited for their partners to start pulling before they themselves decided. Instead, in a majority of trials subjects took the two slices of banana that could have been their leverage or they opened the door to the rotating tray before their partners—an action that could potentially lead to better gains but that, at the same time, also resulted in a loss of their leverage.

Once at this point, the mismatch between the final decisions and the decision latencies raises the question as to what extent chimpanzees acted strategically and whether they did recognize and care about their position of leverage. We have identified two main factors which could have significantly affected the decisions made by the chimpanzees in our study: the time delay and the average gains difference.

We established a ten second delay to separate access to the apparatus by the subject and partner. This measure gave us the possibility to assess whether chimpanzees made decisions in anticipation of others behaviour and not in response to it. It is possible that the ten-second delay was too long (although chimpanzees were familiar with the time delay from the training phase). Such a delay could have increased uncertainty about the possibility to lose rewards. Consequently, to reduce uncertainty and risk, chimpanzees would increase their likelihood to act before their partner had access to the sliding door by choosing the maximum immediate reward available. As a matter of fact, when subjects had no leverage they still pulled before their partners in 65% of trials—even though that was the less demanding situation as subjects did not need to consider the presence of alternative rewards. It is thus possible that even though subjects tried to wait for partners to act—and efficiently did so in 31% of trials—the time delay between each individuals’ access to the apparatus had an influence on their final choices.

The second factor that might have affected chimpanzees’ decisions is the average gains difference. This refers to the average difference between the secure rewards baited on the alternative platform and the rewards on the rotating tray. Although individuals knew how to obtain the three rewards baited on the exterior end of the tray (after extensive experience during the training phase), subjects preferred to access their alternative platform when they were presented with the *leverage* condition (two rewards). It is possible that the difference between the rewards may have been too small (3 vs 2) for chimpanzees to prefer the rewards on the rotating tray (although see [27] for a study showing that apes in this population can distinguish between these amounts of food). Thus, in addition to the increased waiting time, the gain from accessing the rotating tray would potentially only be small.

In addition to the time delay and the average gains difference, chimpanzees might have also preferred to access the alternative platform to reduce the risk of losing rewards: while partners could fail to open the correct door or to pull, subjects only needed to open their access to the alternative platform. However, it is worth mentioning that partners failed to access the rotating tray in only 1.4% of the test trials when they acted after subjects, so with increasing experience in the test, uncertainty about the partner’s actions would reduce.

Results from a different version of the current task supports the interpretation that the combination of the time delay between access to the rewards and the relative difference between gains likely reduces chimpanzees’ probabilities to use their leverage efficiently. In a very recent study using the same apparatus [17] we presented pairs of chimpanzees with simultaneous access to a rotating tray (baited with one reward on the interior end and five rewards on the exterior end) and an alternative platform baited with one reward for each ape. In other words, both chimpanzees had symmetrical options available. As in the current study, chimpanzees could only access one option at a time. We found that chimpanzees preferred to access the alternative platform in approximately 40% of trials. In that study, the average gains difference (i.e. the difference between the average gains from the alternative and the rotating tray) was 1.5 banana slices per trial on the rotating tray. In contrast, in our study, when their leverage consisted of two banana slices (i.e. the true leverage condition) the extra gain from waiting for their partners to pull was of just one banana slice on the rotating tray per trial. Thus, it is likely that the combination of delay between individuals’ access to the apparatus and the limited potential gain from waiting for their partners to act, decreased their likelihood to access the rotating tray. These results are also consistent with a study showing that given the same rewards, chimpanzees prefer to feed alone rather than with conspecifics [28].

Given these results we propose that the chimpanzees solve this conflict of interest through an evaluation of the relevant costs and benefits. When costs are high (for instance in the form of increased delay and uncertainty to get rewards or due to low extra gains from choosing the riskier option), individuals might prevent losses by behaving more conservatively (e.g. choosing a secure option). When costs are lower, individuals might engage in riskier endeavors (e.g. waiting longer for the partner to pull). This framework can also help us to explain the apparent difference between our results and a previous study on chimpanzees´ negotiation abilities during conflict situations [21]. In their study Melis and colleagues [21] found that low-ranking chimpanzees sometimes refused to collaborate with dominants in an obligate cooperative task if the subordinates had the option to access better payoffs for themselves. In such a situation, subordinates would wait until dominants changed their position to cooperate towards the equitable outcome—the one maximizing subordinates’ payoffs. These results can be interpreted as subordinates effectively using the dominant’s need of their help as a way to exert leverage over the dominant’s decisions. However, contrary to the current study, the chimpanzees were obligated to reach a consensus to acquire any rewards, independent action was not possible, and the time pressure to cooperate was lower. In addition, subordinate chimpanzees did not need to anticipate the choices if others to exert pressure and the relative different in gains between the rewards for subordinates was high (1 banana slice vs a whole banana). Thus, the relative benefits of waiting were potentially quite high.

We suggest that the decisions of the chimpanzees in the current experiment were influenced by their evaluation of the risks and uncertainties to obtain the rewards associated with each decision, as well as by their ability to inhibit action. However, we know little about these factors influence on decisions in social contexts. Previous studies have shown that chimpanzees are sensitive to increased risk and uncertainty, but these studies have mainly focused on apes’ unilateral decisions in non-social scenarios [29, 30] with the exception of a study by Rosati and Hare [31]. Their study presented chimpanzees with a choice between a safe and an uncertain outcome that could either be worse or better than the safe one. Chimpanzees preferred the riskier option in a competitive condition where a human experimenter pulled away the option that the apes were approaching. However, risks in this and other tasks [29, 30] come from the assigned probabilities of occurrence for each possible outcome. In contrast, in our task risks are embedded in apes’ conflict of interest. It is possible that social scenarios in which individuals do not possess complete control of the situation (i.e. higher uncertainty) lead individuals towards more conservative decisions. Similarly, some studies have shown that chimpanzees and other primates such as capuchins (*Sapajus apella*) can wait more than 20 seconds (and even up to several minutes) to obtain preferred rewards while refusing less valuable options [32, 33]. Chimpanzees can also wait for partners to engage in mutual collaboration [34] but competitive scenarios, such as the one presented here, may hinder the ability to inhibit prepotent responses: chimpanzees need to divide their attention across different inputs within a limited amount of time (e.g. the payoffs of the game, their partner’s potential actions), resulting in an increased likelihood to opt out for less rewarding but secure outcome to reduce uncertainty and competition. Supporting this interpretation, a recent study shows that the mere presence of another chimpanzee while co-feeding, accelerated chimpanzees feeding rate relative to individual feeding [35].

In conclusion, our study investigated whether chimpanzees would use a position of leverage to maximize their own benefits in a conflict of interest. Chimpanzees did not use their leverage strategically; however, they showed some understanding of the conflict presented and the potential use of their leverage by waiting longer to act during test trials. Possibly, a combination of low extra gains and high risk for waiting led chimpanzees to select their own leverage in a majority of trials.

In the future, novel setups may shed more light on whether chimpanzees use leverage positions for their own benefits. For instance, providing food options differing in quality rather than quantity might result in better self-control and thus a better opportunity to use their leverage efficiently [36, 37]. In addition, chimpanzees could be offered the possibility to interact with different partners varying in their leverage. While controlling for other factors such as social rank, familiarity or sex, strategic individuals would prefer to interact with the partner in possession of the smallest leverage—with whom their interactions would be more symmetrical. In addition, the manipulation of factors we have suggested to be key to their decisions in the current experiment, such as the time delay between responses and the potential benefits from using the leverage correctly, could help disentangle the mechanisms underlying decision-making strategies in chimpanzees as well as other ape species. For instance, bonobos have been shown to be more risk averse than chimpanzees in non-social situations [29, 30] but more risk prone in social settings [38].

## Supporting information

S1 File Supporting information. (DOCX)
