# Supplementary material for: Chimpanzees’ understanding of social leverage
Source: PLoS One. 2018 Dec 12;13(12):e0207868. doi: 10.1371/journal.pone.0207868 (PMC6291185; doi:10.1371/journal.pone.0207868)
Supplement: S1 File — (DOCX) [file pone.0207868.s001.docx]

**Chimpanzees’ leverage understanding**

**Supporting Information**

Subjects

Table A: Subject information

| **Name** | **Sex** | **Age during stud time** | **Paired with (phase 1)** | **Paired with (phase 2)** | **Rearing history** |
| --- | --- | --- | --- | --- | --- |
| Frodo | Male | 24 | Bangolo | Lome | Born in captivity/ mother |
| Lome | Male | 16 | Kofi | Lobo | Born in captivity/ mother |
| Kofi | Male | 12 | Lome | Lobo | Born in captivity/ mother |
| Lobo | Male | 13 | Tai | Kofi | Born in captivity/ mother |
| Taï | Female | 15 | Lobo | Bangolo | Born in captivity/ mother |
| Bangolo | Male | 8 | Frodo | Tai | Born in captivity/ mother |

Model analyses

To implement the Model 1 in R statistics (version 3.1.1) we used the “lme4” package. To obtain the p-values for the individual fixed effects we conducted likelihood-ratio tests. We assessed the model stability by comparing the estimates derived from a model based on all data with those obtained from models with the levels of the random effects excluded one at a time. The model was stable.

To implement Models 2 to 4 we used the “coxme” function from the “coxme” package (Therneau 2012). This approach allows to analyse the variability attributable to the independent variables while controlling for right-censored data (i.e. when individuals did not act after a limited time established by the experimenter). Cox proportional hazards models without random effects (implemented with the “coxph” function from the “survival” package) were used to create the plots.

To rule out collinearity we checked the variance inflation factors (VIF) for the GLMM and the coxme models. All VIF values were closer to 1.

Inter-observer reliability

The inter-observer reliability was excellent based on the 20% of the data (subjects’ opening the sliding door: Cohen Kappa = 0.94; partners’ opening the sliding door: Cohen Kappa = 1; subjects’ pulling the rope: Cohen Kappa = 0.95; partners’ pulling the rope: Cohen Kappa = 1; subjects’ accessing the leverage: Cohen Kappa = 0.96). To calculate the inter-observer agreement of the latency data, we only considered trials in which both observers scored the actions that led to latencies (either to open a door or to pull). The inter-observer reliability based on the 20% of the data was excellent (subjects’ and partners’ latency to open the access to the social tray: r = 0.99; subjects’ and partners’ latency to pull the rope: r ≥ 0.97; subjects’ latency to open the access to the leverage: r = 0.99)

Model 1: Choices of subjects accessing first

Model 1 investigated whether subjects accessing the apparatus before their partners would use the leverage strategically for their own benefit. We hypothesize that if subjects understand the potential use of the leverage baited on the alternative platform, they would access the level two leverage more often in control compared to test trials. In the latter condition they would wait for their partner to pull and retrieve the rewards baited on the exterior end of the rotating tray. The full model included the test variables condition and leverage level as well as the interaction between them. The control variables were trial, session, phase, the role of the pair members and the sex of the dyad as fixed effects; dyad, individual on the right side and individual on the left side as random effects and the random slopes. The comparison between the full and the null model was significant (GLMM: *χ*^2^ = 16.44, df = 3, p<0.001, N = 288). We dropped the non-significant two-way interaction between condition*leverage level (GLMM, *χ*^2^_1_ = 0.08, p = 0.77, N = 288). We found a main effect of leverage (see Table 2). During test and control trials, chimpanzee subjects significantly accessed their alternative platform more often than the rotating tray when the former was baited with leverage level two or four. In contrast, chimpanzees preferred to access the rotating tray when no food was baited on the alternative platform (leverage level zero).

Table 2: Model 1 information

| Term | Estimate | Standard Error | Chi-square | Degrees of freedom | p-value | CI (95%) of the model |
| --- | --- | --- | --- | --- | --- | --- |
| Intercept | 4.31 | 1.07 | - | - | - | 2.4, 84.5 |
| Condition | -0.74 | 0.68 | 1.14 | 1 | 0.29 | -27.4, 10.9 |
| Leverage | 7.09 | 1.63 | 14.24 | 1 | <0.001 | 4.2, 145.6 |
| Session | 0.61 | 0.37 | 3.06 | 1 | 0.08 | -3.7, 16.6 |
| Trial | -0.99 | 0.42 | 4.83 | 1 | 0.028 | -24.8, 0.31 |
| Phase | 0.57 | 0.6 | 0.91 | 1 | 0.34 | -3.8, 16.6 |
| Role pair | -0.22 | 0.71 | 0.1 | 1 | 0.75 | -13.9, 11.9 |
| Dyad sex | - | - | 1.48 | 1 | 0.22 | - |

Model 2: Subjects latency to open the door when acting first.

Model 2 investigated the latencies of subjects to access the apparatus when they acted before their partners. We hypothesized that, if subjects understand the potential use of the leverage baited on the alternative platform, they would wait longer to access the apparatus when this leverage consisted of zero or two pieces of food instead of four. In addition, we expected the overall latencies to be higher in test compared to control trials. For this model we established a censor to account for trials in which subjects did not open the door after 20 seconds and for trials in which partners acted before them. The censored data represented 5.6% of the total data (16 of 288 trials). The full model included the test variables condition and level of leverage as well as the interaction between them. The control variables were trial, session, phase, the role of the pair members and the sex of the dyad as fixed effects and dyad, subject identity and partner identity as random effects. The comparison between the full and the null model was significant (coxme, *χ*^2^  = 23.44, df = 3, p<0.001, N = 288). We found a significant two-way interaction between condition*leverage level (coxme, HR = 1.45, p = 0.004, N = 288, CI = 1.12, 1.87). Individuals waited longer to open the sliding door the smaller the leverage was and this effect was more pronounced in test compared to control trials (see Table 3).

Table 3: Model 2 information

| Term | Hazard Rate (HR) | p-value | CI (95%) of the model |
| --- | --- | --- | --- |
| Condition (test) | 1.03 | 0.83 | 0.78, 1.35 |
| Leverage | 1.09 | 0.35 | 0.91, 1.31 |
| Session | 0.96 | 0.58 | 0.85, 1.05 |
| Trial | 0.93 | 0.26 | 0.82, 1.86 |
| Phase | 1.04 | 0.46 | 0.92, 1.19 |
| Role pair | 0.97 | 0.69 | 0.84, 1.12 |
| Dyad sex | - | 0.32 | - |
| Condition *Leverage | 1.45 | 0.0043 | 1.12, 1.87 |

Model 3: Latency of subjects to pull their rope when acting first.

Model 3 investigated subjects´ latencies to pull once they had already opened the access to the rotating tray. We hypothesize that if subjects understood the conflict of interest presented, they would wait longer to pull in test compared to control trials. In addition, we investigated whether the presence of the leverage baited on the alternative platform affected subjects’ latencies to pull—at this point the leverage was no longer accessible but still visible. For this model we established a censor to account for trials in which subjects did not open the door and trials in which partners pulled before them. The censored data represented 18.8% of the total data (19 of 101 trials). The full model included the test variables condition and leverage. The control variables were trial, session, phase, the role of the pair members and the sex of the dyad as fixed effects and dyad, subject and partner as random effects. The comparison between the full and the null model was significant (coxme, *χ*^2^ = 11.99, df = 2, p = 0.002, N = 101). We found a main effect of condition suggesting that subjects waited longer to pull in test compared to control trials. We found no significant effect of leverage (see Table 4).

Table 4: Model 3 information

| Term | Hazard rate (HR) | p-value | CI (95%) of the model |
| --- | --- | --- | --- |
| Condition (test) | 0.36 | <0.001 | 0.22, 0.58 |
| Leverage | 1.11 | 0.41 | 0.87, 1.41 |
| Session | 0.89 | 0.34 | 0.7, 1.13 |
| Trial | 0.97 | 0.79 | 0.77, 1.22 |
| Phase | 1.31 | 0.03 | 1.02, 1.67 |
| Role pair | 1.06 | 0.7 | 0.79, 1.42 |
| Dyad sex | - | 0.11 | - |

Model 4: Partners’ latency to pull their rope when acting first.

Model 4 investigated partners´ latencies to act from the moment they could access the sliding door until they pulled from their rope. The model excluded when partners´ mistakenly accessed their alternative platform (*N* = 2). We hypothesize that, if partners understood the conflict of interest presented, they would wait longer to pull in test compared to control trials and this effect would be more pronounced the lower the leverage was (either level zero or two). The censored-data included those trials in which partners did not pull before the time limit of 20 seconds and those trials in which subjects pulled before them. The censored data represented 23.6 % of the total data (66 of 280 trials). The full model included the test variables condition and level of leverage as well as the interaction between them. The control variables were trial, session, phase, the role of the pair members and the sex of the dyad as fixed effects and dyad, subject ID and partner ID as random effects. The comparison between the full and the null model was almost significant (coxme, *χ*^2^ = 7.75, df = 3, p = 0.051, N = 280). We dropped the non-significant two-way interaction between condition and leverage level (coxme, HR = 0.85, p = 0.27, N = 280). We found a main effect of condition suggesting that partners waited longer to pull in test compared to control trials. We found no significant effect of leverage (see Table 5).

Table 5: Model 4 information

| Term | Hazard rate (HR) | p-value | CI (95%) of the full model |
| --- | --- | --- | --- |
| Condition (test) | 0.47 | 0.002 | 0.29, 0.76 |
| Leverage | 0.98 | 0.74 | 0.84, 1.12 |
| Session | 1.12 | 0.13 | 0.97, 1.3 |
| Trial | 1.04 | 0.57 | 0.9, 1.2 |
| Phase | 1.1 | 0.25 | 0.94, 1.29 |
| Role pair | 0.96 | 0.62 | 0.8, 1.14 |
| Dyad sex (mix) | - | 0.34 | - |
